# Peakhood: individual site context extraction for CLIP-seq peak regions

**DOI:** 10.1093/bioinformatics/btab755

**Published:** 2021-11-04

**Authors:** Michael Uhl, Dominik Rabsch, Florian Eggenhofer, Rolf Backofen

**Affiliations:** Bioinformatics Group, Department of Computer Science, University of Freiburg, Freiburg im Breisgau, Germany; Bioinformatics Group, Department of Computer Science, University of Freiburg, Freiburg im Breisgau, Germany; Bioinformatics Group, Department of Computer Science, University of Freiburg, Freiburg im Breisgau, Germany; Bioinformatics Group, Department of Computer Science, University of Freiburg, Freiburg im Breisgau, Germany; Signalling Research Centres BIOSS and CIBSS, University of Freiburg, Freiburg im Breisgau, Germany

## Abstract

**Motivation:**

CLIP-seq is by far the most widely used method to determine transcriptome-wide binding sites of RNA-binding proteins (RBPs). The binding site locations are identified from CLIP-seq read data by tools termed peak callers. Many RBPs bind to a spliced RNA (i.e. transcript) context, but all currently available peak callers only consider and report the genomic context. To accurately model protein binding behavior, a tool is needed for the individual context assignment to CLIP-seq peak regions.

**Results:**

Here we present Peakhood, the first tool that utilizes CLIP-seq peak regions identified by peak callers, in tandem with CLIP-seq read information and genomic annotations, to determine which context applies, individually for each peak region. For sites assigned to transcript context, it further determines the most likely splice variant, and merges results for any number of datasets to obtain a comprehensive collection of transcript context binding sites.

**Availability and implementation:**

Peakhood is freely available under MIT license at: https://github.com/BackofenLab/Peakhood.

**Supplementary information:**

Supplementary data are available at *Bioinformatics* online.

## 1 Introduction

CLIP-seq (cross-linking and immunoprecipitation followed by next generation sequencing) (Licatalosi *et al.*, 2008) is the most widely used procedure to experimentally determine the exact transcriptome-wide binding locations of RNA-binding proteins (RBPs). The most popular protocol variants are PAR-CLIP (Hafner *et al.*, 2010), iCLIP (König *et al.*, 2010) and eCLIP (Van Nostrand *et al.*, 2016). CLIP-seq is usually performed *in vivo* for a specific RBP, resulting in a library of reads bound by the target RBP. Binding sites are subsequently identified by mapping the reads back to the reference genome, and analyzing the read profiles with tools referred to as peak callers. A number of peak callers have been popular over the years, such as Piranha (Uren *et al.*, 2012), CLIPper (Lovci *et al.*, 2013) or PureCLIP (Krakau *et al.*, 2017).

Calling peaks in the genomic context, as done by all currently available peak callers, is unbiased for RBPs that predominantly bind to unspliced RNA. However, for RBPs that predominantly bind in a spliced (i.e. transcript) context, this is clearly suboptimal. Indeed, a recent study (Uhl *et al.*, 2020) has demonstrated this to be a substantial problem, and that the inclusion of transcript context can improve the identification of authentic binding sites. Peak callers applied to CLIP-seq data have produced millions of publicly available binding sites, e.g. from ENCODE (Van Nostrand *et al.*, 2020b). Consequently, a tool is required that can analyze CLIP-seq peak regions to extract the individual site context for each peak region.

Here, we present Peakhood, the first tool capable of extracting the most likely site context, individually for each CLIP-seq peak region. The necessary information are extracted directly from the CLIP-seq read profiles, in combination with a genomic annotations file (both reference and custom annotations are supported). For sites assigned to transcript context, Peakhood further determines the most likely splice variant. In addition, Peakhood can merge extracted transcript context sets into comprehensive transcript context site collections. Peakhood also supports batch processing, i.e. context extraction of multiple datasets and merging in one run. As a supplement, we provide four precomputed transcript context site collections, using eCLIP datasets of 49 RBPs with known roles in posttranscriptional gene regulation (see Data availability section).

## 2 Approach

**Fig. 1.**
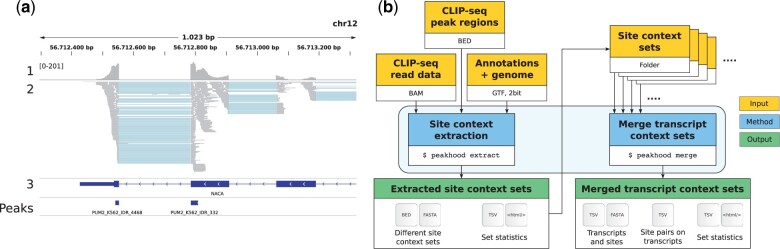
(**a**) Genomic region (IGV screenshot) with mapped PUM2 K562 eCLIP data (details in Supplementary Section 1.4). 1: Read profile (coverage range in brackets), 2: read alignments, 3: gene annotations (thick blue regions are exons, thin blue regions introns), Peaks: peaks called by CLIPper IDR method (high-confidence peaks reproducible between replicates). Example transcript context region for the predominantly spliced RNA-binding RBP PUM2, where an exon border site is falsely split in two peaks. (**b**) Overview of the Peakhood workflow for the two main program modes extract and merge. Yellow boxes mark necessary inputs, blue boxes the two program modes and green boxes the outputs. Arrows show the dependencies between inputs, modes and outputs

Here, we briefly describe how Peakhood works. A detailed description can be found in the Online Supplementary (Section 1.2). For full details, please check out the comprehensive manual on GitHub. Peakhood first extracts the site context for each input peak region. Figure  1a shows two peak regions inside a typical transcript context. Peakhood uses the given exon annotations (GTF) and CLIP-seq read information (BAM), essentially looking for differences in exon and surrounding intron coverage, as well as coverage drops at exon borders. If these differences exceed the configured thresholds, the site is assigned to transcript context, otherwise to genomic context (Supplementary Fig. S1 example). In addition, sites at exon borders connected by intron-spanning reads are merged into single sites (as in Fig.  1a). For sites assigned to transcript context, Peakhood further selects the most likely site-transcript combination, using various read, site and transcript statistics. Moreover, Peakhood can merge single datasets into comprehensive transcript context site collections (see Fig.  1b for the extraction and merge workflow). The collections also include tabular data, e.g. to identify which sites on transcripts are in close distance, or if site distances decreased compared to the original genomic context. Percentages of extracted transcript context sites agree with known RBP roles (see Supplementary Section 1.3 and Fig. S2). Peakhood requires a Linux operating system and is easy to install, e.g. via Conda (Conda package available). The tool was tested (Intel i7-8700k, Ubuntu 18.04 LTS), with single dataset site context extraction (example dataset with 2146 input peak regions, see Supplementary Section 1.6) taking about 2 min and 30 s.

## 3 Conclusion

Here we presented Peakhood, the first tool capable of extracting the most likely site context, individually for each CLIP-seq peak region. Peakhood is easy to install and use, thanks to its comprehensive online manual, and it works with standardized file formats (BAM, BED, GTF, 2 bit). We demonstrated Peakhood’s capabilities with eCLIP data and peak regions obtained from ENCODE (Van Nostrand *et al.*, 2020b). However, it is not limited to this type of data, and should work fine with other HTS peak data (iCLIP, PAR-CLIP, OOPS), as well as other peak caller outputs, e.g. from PureCLIP. The flexibility is further increased through Peakhood’s various command line parameters, to adapt it for individual datasets or new input types. Summing up, Peakhood allows for an improved modeling of protein binding behavior, by providing a more authentic sequence and structure context, especially for spliced RNA-binding proteins. 

## Supplementary Material

btab755_supplementary_data
