# Supplementary material for: Peakhood: individual site context extraction for CLIP-seq peak regions
Source: Bioinformatics. 2021 Nov 4;38(4):1139–40. doi: 10.1093/bioinformatics/btab755 (PMC8796379; doi:10.1093/bioinformatics/btab755)
Supplement: btab755_supplementary_data [file btab755_supplementary_data.pdf]

Peakhood: individual site context extraction for CLIP-seq peak regions
Supplementary Material

Michael Uhl, Dominik Rabsch, Florian Eggenhofer, and Rolf Backofen

October 28, 2021

1 Supplementary methods

1.1 Data availability

The transcript context site collections generated by Peakhood from eCLIP datasets of 49 RBPs (first collection with 36 RBPs from HepG2, second collection with 40 RBPs from K562) with known roles in post-transcriptional gene regulation (mRNA stability and decay, translational regulation; information taken from [1] Supplementary Data 1 table) can be downloaded from Zenodo [2].

1.2 How Peakhood works

Here we briefly describe how Peakhood works. For full details, please check out Peakhood's comprehensive online manual at: https://github.com/BackofenLab/Peakhood

Site context extraction

To extract individual site context information for a CLIP-seq dataset, Peakhood's input consists of the genomic CLIP-seq peak regions (BED), the mapped CLIP-seq reads (BAM), a genomic annotations file (GTF), and a genome sequence file (.2bit). Peakhood first intersects the peak regions with transcript and exon regions from the GTF file, to obtain exonic, intronic, and intergenic sites. Next it determines for each exonic site whether it is more likely embedded in a genomic context (introns included) or transcript context (mature or spliced RNA). For this Peakhood makes use of the exon-intron read coverage ratios in the site neighborhood, as well as over the whole transcript. This is based on the observation that an exonic site inside a transcript (spliced) context (Paper Fig. 1a) usually features considerably more reads in the exon region(s), as well as a pronounced coverage drop-off at the exon borders. Ideally this is true both locally (around the overlapping exon) and globally (on the whole transcript). However, due to how the CLIP-seq protocol works, read coverage is often concentrated at and around the binding site, so Peakhood weighs the local context information higher than the global one. In addition, intron-spanning reads receive more weight than continuously mapped reads, since they provide strong support for a transcript context. Sites which feature sufficiently high local

and global ratios (see online manual for more details on filter steps and thresholds) get assigned to transcript context. Exonic sites with lower ratios get assigned to genomic context (Fig. S1).

Choosing the most likely transcript

Since a gene usually has many transcript isoforms, and there can be several overlapping exons and transcripts which pass the filters, a transcript context site can have several possible site-transcript combinations. `Peakhood` thus also selects the most likely combination, based on number of informative filters: co-occurrence of other sites, read coverage, intron-spanning read numbers, and transcript support level. Filter order, choice of filters, and filter behavior (serial or majority vote) can be further customized. In addition, sites at exon borders connected by intron-spanning reads get merged into single sites (see Paper Fig. 1a example). Reference and custom transcript annotations are supported, which can be advantageous if created for the same cell types or conditions (see online manual on how to create custom annotations). Incorporation of RNA-seq data is also possible, to provide additional intron-spanning read information for transcript selection.

Merging transcript context sets

In addition to extracting transcript context sites for single CLIP-seq datasets, `Peakhood` can merge any number of transcript context sets into comprehensive transcript context site collections (see Paper Fig. 1b for general workflow). Output table files contain information on transcripts and overlapping sites, both for all and the most likely site-transcript combinations. Site pairs on transcripts and their genomic and transcript distances are also reported. This way, one e.g. can quickly filter for and spot interesting site pairs (same or different RBP), where the transcript site distance is lower than the original genomic distance.

1.3 Agreement with known RBP roles

When `Peakhood` performs the site context extraction, it reports (among other statistics) three informative percentages: the percentage of exonic sites (divided by all sites), the percentage of transcript context sites (divided by all exonic sites), and the percentage of exon border sites (divided by all transcript context sites). Fig. S2 shows these percentages for four eCLIP datasets from four different RBPs, obtained by running site context extraction (`peakhood extract`) with default parameters. We can see that for typical spliced RNA binding RBPs (IGF2BP1, PUM1, PUM2), most sites overlap with exons (>= 95%), and out of these >= 95% are assigned to transcript context. In contrast, for the splicing factor U2AF2 we get around 20% of exonic sites, and out of these only 5.9% get assigned to transcript context. This shows that `Peakhood`'s transcript context selection agrees with known RBP roles. We also see that the number of exon border sites can be quite substantial, as in the case of PUM1 (around 25%). Such sites at exon borders connected by intron-spanning reads need to be merged, and not taken as separate binding events (see Paper Fig. 1). This again showcases the importance of a proper site context selection as done by `Peakhood`.

1.4 Displaying genomic regions

To display the genomic regions in Figure 1a and Figure S1, BAM and IDR peak files from ENCODE were downloaded (dataset IDs ENCSR661ICQ (PUM2) and ENCSR893RAV (U2AF2)) and pre-processed as follows:

```
wget https://www.encodeproject.org/files/ENCFF880MWQ/@@download/ENCFF880MWQ.bed.gz
gunzip -c ENCFF880MWQ.bed.gz | awk '{print $1"\t"$2"\t"$3"\t"$4"\t"$7"\t"$6}' > PUM2_K562_IDR_peaks.bed
wget -O PUM2_K562_rep1.bam https://www.encodeproject.org/files/ENCFF231WHF/@@download/ENCFF231WHF.bam
wget -O PUM2_K562_rep2.bam https://www.encodeproject.org/files/ENCFF732EQX/@@download/ENCFF732EQX.bam
samtools merge -f PUM2_K562_rep12.bam PUM2_K562_rep1.bam PUM2_K562_rep2.bam
samtools view -hb -f 130 PUM2_K562_rep12.bam -o PUM2_K562_rep12.R2.bam
wget https://www.encodeproject.org/files/ENCFF290DFO/@@download/ENCFF290DFO.bed.gz
gunzip -c ENCFF290DFO.bed.gz | awk '{print $1"\t"$2"\t"$3"\t"$4"\t"$7"\t"$6}' > U2AF2_K562_IDR_peaks.bed
wget -O U2AF2_K562_rep1.bam https://www.encodeproject.org/files/ENCFF835KXL/@@download/ENCFF835KXL.bam
wget -O U2AF2_K562_rep2.bam https://www.encodeproject.org/files/ENCFF936JSP/@@download/ENCFF936JSP.bam
samtools merge -f U2AF2_K562_rep12.bam U2AF2_K562_rep1.bam U2AF2_K562_rep2.bam
samtools view -hb -f 130 U2AF2_K562_rep12.bam -o U2AF2_K562_rep12.R2.bam
```

The merged R2 read BAM and peak region BED files were then loaded up into IGV (Integrative Genome Viewer) for visualization [link].

1.5 Site context extraction percentages

To get the percentages of Figure S2, we used a custom GTF file generated as described in the online manual (see "Documentation" section, subsection "Custom GTF files", total RNA-seq ENCODE dataset ID: ENCSR792OIJ). The created custom GTF file for the K562 cell line can be downloaded from Zenodo [2]. The BAM and BED files were downloaded and pre-processed as described in the below section "Runtime measurement", encompassing the following four ENCODE eCLIP datasets: ENCSR975KIR (IGF2BP1), ENCSR308YNT (PUM1), ENCSR661ICQ (PUM2), and ENCSR893RAV (U2AF2). Using the custom GTF file, site context extraction was evoked to get the percentages from Figure S2 by the following commands:

```
peakhood extract --in IGF2BP1_K562_IDR_peaks.uniq_ids.bed --bam IGF2BP1_K562_rep12.R2.bam
--gtf K562_total_rnaseq_rep12_stringtie_gffcompare.gtf --gen hg38.2bit --out IGF2BP1_K562_IDR_pm_extract_out
--pre-merge --new-site-id IGF2BP1_K562_IDR
peakhood extract --in PUM1_K562_IDR_peaks.uniq_ids.bed --bam PUM1_K562_rep12.R2.bam
--gtf K562_total_rnaseq_rep12_stringtie_gffcompare.gtf --gen hg38.2bit --out PUM1_K562_IDR_pm_extract_out
--pre-merge --new-site-id PUM1_K562_IDR
peakhood extract --in PUM2_K562_IDR_peaks.uniq_ids.bed --bam PUM2_K562_rep12.R2.bam
--gtf K562_total_rnaseq_rep12_stringtie_gffcompare.gtf --gen hg38.2bit --out PUM2_K562_IDR_pm_extract_out
--pre-merge --new-site-id PUM2_K562_IDR
peakhood extract --in IGF2BP1_K562_IDR_peaks.uniq_ids.bed --bam U2AF2_K562_rep12.R2.bam
--gtf K562_total_rnaseq_rep12_stringtie_gffcompare.gtf --gen hg38.2bit --out U2AF2_K562_IDR_pm_extract_out
--pre-merge --new-site-id U2AF2_K562_IDR
```

1.6 Runtime measurement

For the runtime measurement (inside conda environment with `Peakhood` installed), we downloaded and pre-processed the eCLIP PUM1 data (K562 cell line) as described in `Peakhood`'s online manual on GitHub [3].

```
wget https://www.encodeproject.org/files/ENCFF094MQV/@@download/ENCFF094MQV.bed.gz
gunzip -c ENCFF094MQV.bed.gz | awk '{print $1"\t"$2"\t"$3"\t"$4"\t"$7"\t"$6}' > PUM1_K562_IDR_peaks.bed
bed_generate_unique_ids.py  --in PUM1_K562_IDR_peaks.bed --id PUM1_K562_IDR > PUM1_K562_IDR_peaks.uniq_ids.bed
wget -O PUM1_K562_rep1.bam https://www.encodeproject.org/files/ENCFF064COB/@@download/ENCFF064COB.bam
```

```
wget -O PUM1_K562_rep2.bam https://www.encodeproject.org/files/ENCFF583QFB/@@download/ENCFF583QFB.bam
wget https://hgdownload.cse.ucsc.edu/goldenpath/hg38/bigZips/hg38.2bit
wget http://ftp.ensembl.org/pub/release-103/gtf/homo_sapiens/Homo_sapiens.GRCh38.103.gtf.gz
```

Now we can run the site context extraction on the dataset:

```
peakhood extract --in PUM1_K562_IDR_peaks.uniq_ids.bed --bam PUM1_K562_rep1.bam PUM1_K562_rep2.bam
--bam-pp-mode 2 --gtf Homo_sapiens.GRCh38.103.gtf.gz --gen hg38.2bit --report
--out PUM1_K562_IDR_extract_out

peakhood extract --in PUM1_K562_IDR_peaks.uniq_ids.bed --bam PUM1_K562_rep1.bam PUM1_K562_rep2.bam
--bam-pp-mode 2 --gtf Homo_sapiens.GRCh38.103.gtf.gz --gen hg38.2bit --out PUM1_K562_IDR_pm_extract_out
--pre-merge --new-site-id PUM1_K562_IDR
```

On our test machine (Intel i7-8700k, 32 GB RAM, Ubuntu 18.04 LTS), this takes about 2 minutes and 30 seconds. In case the dataset is used more than once, we recommend to pre-merge the BAM files, as well as filter by R2 reads (in case of eCLIP data, as described in online manual), to speed up the run:

```
samtools merge -f PUM1_K562_rep12.bam PUM1_K562_rep1.bam PUM1_K562_rep2.bam
samtools view -hb -f 130 PUM1_K562_rep12.bam -o PUM1_K562_rep12.R2.bam
```

Running this again shortens to extraction time to about 1 minute and 30 seconds:

```
peakhood extract --in PUM1_K562_IDR_peaks.uniq_ids.bed --bam PUM1_K562_rep12.R2.bam
--gtf Homo_sapiens.GRCh38.103.gtf.gz --gen hg38.2bit --out PUM1_K562_IDR_pm_extract_out
--pre-merge --new-site-id PUM1_K562_IDR
```

Supplementary figures

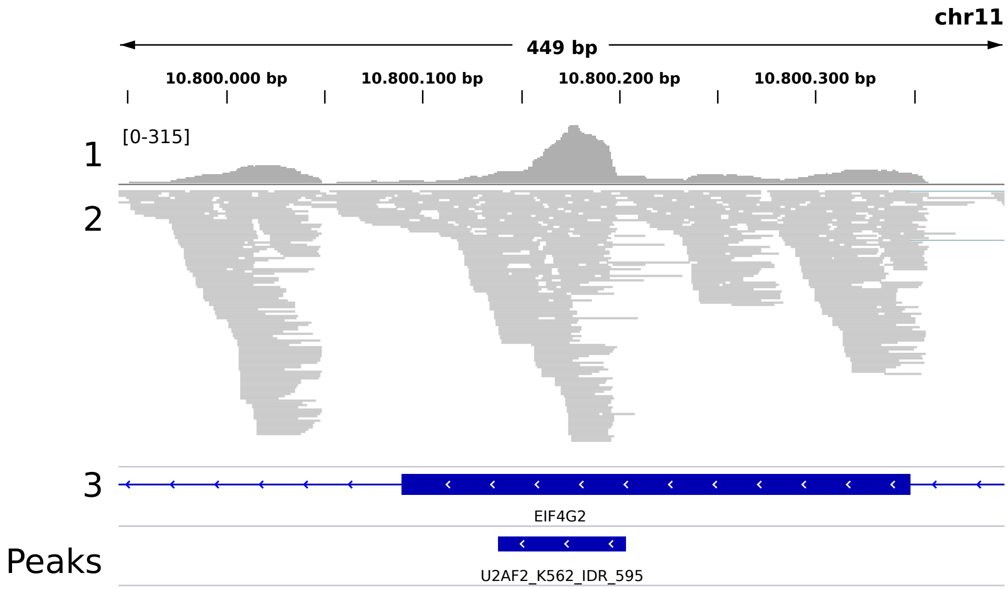

Figure 1: Genomic region (IGV snapshot) with mapped U2AF2 eCLIP data. 1: read profile (coverage range in brackets), 2: read alignments, 3: gene annotations (thick blue regions are exons, thin blue regions introns), Peaks: peaks called by CLIPper (IDR method). Example region for the splicing factor U2AF2, with higher read counts over exon borders and introns.

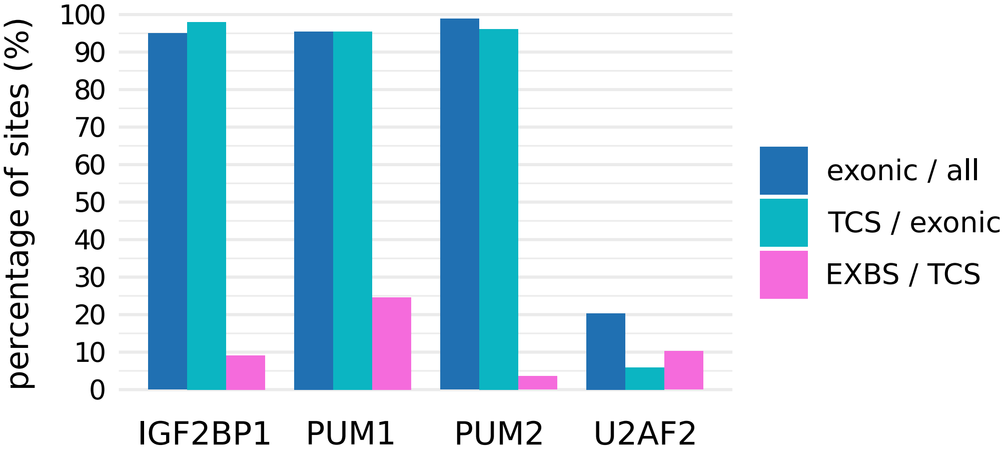

Figure 2: `Peakhood` site extraction results for four eCLIP datasets (K562 cell line, sites from CLIPper IDR) and four RBPs (number of all sites in brackets): IGF2BP1 (4776), PUM1 (2146), PUM2 (4578), and U2AF2 (3250). The plot shows percentages of exonic sites (exonic sites divided by all sites), transcript context sites (TCS) (TCS divided by all exonic sites), and exon border sites connected by intron-spanning reads (EXBS) (EXBS divided by TCS).

References

[1] Eric L Van Nostrand, Peter Freese, Gabriel A Pratt, Xiaofeng Wang, Xintao Wei, Rui Xiao, Steven M Blue, Jia-Yu Chen, Neal AL Cody, Daniel Dominguez, et al. A large-scale binding and functional map of human RNA-binding proteins. *Nature*, 583(7818):711–719, 2020.

[2] Michael Uhl. Peakhood: individual site context extraction for CLIP-seq peak regions. *Zenodo*, 2021. https://doi.org/10.5281/zenodo.5557101.

[3] Michael Uhl. Peakhood: individual site context extraction for CLIP-seq peak regions. *GitHub repository*, 2021. https://github.com/BackofenLab/Peakhood.
